# Sirt4: A Multifaceted Enzyme at the Crossroads of Mitochondrial Metabolism and Cancer

**DOI:** 10.3389/fonc.2020.00474

**Published:** 2020-04-15

**Authors:** Daniela Tomaselli, Clemens Steegborn, Antonello Mai, Dante Rotili

**Affiliations:** ^1^Department of Chemistry and Technology of Drugs, “Sapienza” University of Rome, Rome, Italy; ^2^Department of Biochemistry, University of Bayreuth, Bayreuth, Germany

**Keywords:** sirtuins, protein deacylation, mitochondria, metabolism, cancer

## Abstract

Sirtuins are NAD^+^-dependent deacylases that play crucial roles in the regulation of cellular metabolism, and as a result, are implicated in several diseases. The mitochondrial sirtuin Sirt4, for a long time considered as mainly a mono-ADP-ribosyltransferase, recently has shown a robust deacylase activity in addition to the already accepted substrate-dependent lipoamidase and deacetylase properties. Through these and likely other enzymatic and non-enzymatic activities, Sirt4 closely controls various metabolic events, and its dysregulation is linked to various aging-related disorders, including type 2 diabetes, cardiac hypertrophy, non-alcoholic fatty liver disease, obesity, and cancer. For its capability to inhibit glutamine catabolism and for the modulation of genome stability in cancer cells in response to different DNA-damaging conditions, Sirt4 is proposed as either a mitochondrial tumor suppressor or a tumor-promoting protein in a context-dependent manner. In addition to what is already known about the roles of Sirt4 in different biological settings, further studies are certainly still needed in order to validate this enzyme as a new potential target for various aging diseases.

## Introduction

Sirt4 is one of the three mitochondrial sirtuins and, despite that it was firstly described as a mono-ADP-ribosyltransferase, nowadays it has demonstrated a robust deacylase activity toward 3-hydroxy-3-methyl-glutarylated (HMG) lysine residues (1) as well as substrate-specific lipoamidase and deacetylase properties (2, 3). Sirt4 directly or indirectly regulates multiple mitochondrial functions closely connected to the progression of age-related diseases such as type 2 diabetes (T2D), neurodegeneration, and cancer (1–6).

## Sirt4 Enzymatic Activities

**Figure 1:**
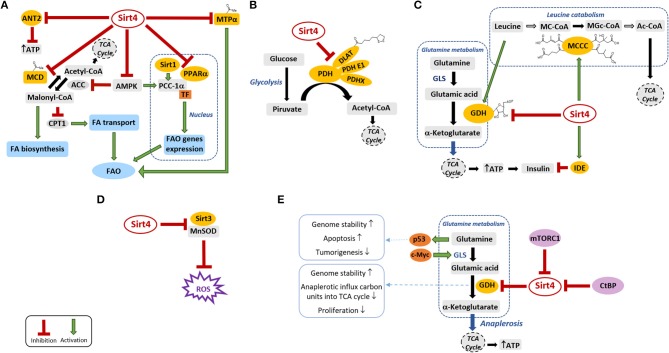
Sirt4 and its substrates in the mitochondrial metabolic pathways. Sirt4 modulates directly or indirectly the activity of several targets (depicted in yellow) that play key roles in various metabolic processes. Green arrows and red lines indicate the promotion/activation and suppression/inhibition of a specific activity, respectively. **(A)** Sirt4 modulation of lipid metabolism. **(B)** Sirt4 inhibition of PDH through lipoamidase activity. **(C)** Sirt4 modulation of insulin secretion and sensitivity. **(D)** Sirt4 effects on mitochondrial oxidative stress. **(E)** Sirt4 and cancer. ACC, acetyl-CoA carboxylase; Ac-CoA, acetyl coenzyme A; AMPK, AMP-activated protein kinase; ANT2, adenine nucleotide translocator 2; ATP, adenosine triphosphate; CPT1, carnitine palmitoyl-transferase 1; CtBP, *C*-terminal binding protein; DLAT, dihydrolipoyllysine acetyltransferase; FA, fatty acids; FAO, fatty acids oxidation; GDH, glutamate dehydrogenase; GLS, glutaminase; IDE, insulin degrading enzyme; MCCC, 3-methylcrotonyl-CoA carboxylase; MC-CoA, 3-methylcrotonyl-CoA; MGc-CoA, 3-methylglutaconyl-CoA; MCD, malonyl-CoA decarboxylase; MnSOD, manganese-dependent superoxide dismutase; mTORC1, mammalian target of rapamycin complex 1; MTPα, mitochondrial trifunctional protein α; PGC-1α, peroxisome proliferator-activated receptor gamma co-activator 1α; PDH, pyruvate dehydrogenase; PPARα, peroxisome proliferator-activated receptors α; ROS, reactive oxygen species; TCA, tri-carboxylic acid; TF, transcription factor.

Sirt4 is distributed in both fetal and adult tissues, with higher expression levels in liver, heart, spleen, prostate, testis, kidney, ovary, white adipose tissue, and muscle, and it works as a “bridge protein” between mitochondrial metabolism and tumorigenesis (7). Several pieces of evidence showed that Sirt4 covers a crucial role when cells are subject to toxic stresses able to modulate the mitochondrial levels of the co-substrate NAD^+^ and/or the NAD^+^/NADH ratio (3–6). As a mono-ADP-ribosyltransferase, Sirt4 catalyzes the transfer of an ADP-ribosyl moiety from NAD^+^ to glutamate dehydrogenase (GDH), thus inhibiting its activity in mice pancreatic β-cells and blocking the anaplerotic influx of carbon units entering the tricarboxylic acids (TCA) cycle (8) (Figure 1). Beyond this capability to regulate energy and glutamine metabolism, an important Sirt4 activity is its lipoamidase-mediated inhibition of pyruvate dehydrogenase (PDH), the crucial multi-component enzymatic complex that modulates the entrance of acetyl-CoA deriving from glycolysis into the TCA cycle, identifying Sirt4 as a “guardian of cellular metabolism” (9). Despite the lack of deacetylase activity on histone peptides *in vitro*, an acetylome peptide microarray study showed a low but substrate-specific and reproducible deacetylase activity for Sirt4 against acetylation sites in, e.g., the mitochondrial proteins Stress-70, NAD(P) transhydrogenase, and Hsp60 (10). Moreover, Sirt4 was also shown to deacetylate and inhibit the activity of malonyl-CoA decarboxylase (MCD) in white adipose tissue and skeletal muscle, thereby regulating fatty acid oxidation (FAO) and biosynthesis processes (11) (Figure 1). Recently, the mitochondrial trifunctional protein α-subunit (MTPα) has also been identified as a substrate of Sirt4 deacetylation in hepatocytes, highlighting the capability of this enzyme to inhibit FAO also in the liver (12) (Figure 1).

In line with its little to no-detectable ADP-ribosyltransferase and deacetylase activities *in vitro* (13, 14), the difficulty to purify the enzyme and the consequent impossibility to set up an efficient enzymatic assay represented for years the main obstacles in the investigation of Sirt4 functions and are the principal reasons for the absence of specific Sirt4 modulators up to now. In 2017, we reported for Sirt4 an efficient deacylase activity toward HMG-modified lysine residues of peptides and proteins and established a fluorescence-based Fluor de Lys-like assay for the screening of large numbers of potential modulators (1). We further solved the crystal structure of Sirt4 from *Xenopus tropicalis* in complex with ADP-ribose, revealing two main isoform-specific features: an extra-active site entry channel connected to the protein surface and a large, dynamic loop reaching the active site, which may contribute to substrate binding and regulate active site dynamics, thus explaining the broad acyl selectivity of Sirt4 (1).

Indeed in the same year Anderson *et al*. showed that Sirt4 removes HMG and related acyl moieties such as glutaryl (G), 3-methylglutaryl (MG), and 3-methylglutaconyl (MGc) from the lysine residues of various substrates both *in vivo* and *in vitro* (15). Among them, an important metabolic substrate is the enzyme complex methylcrotonyl-CoA carboxylase (MCC) that catalyzes the conversion of 3-methylcrotonyl-CoA (MC) into MGc-CoA and plays a crucial role in leucine catabolism. Important metabolic intermediates in this process are also HMG-CoA and MG-CoA (16) that, together with G-CoA, MGc-CoA, and other acyl-CoA, seem able to spontaneously acylate and destabilize the MCC itself, reducing leucine flux through the branched chain amino acid catabolic pathway (15) (Figure 1). In this context, the broad-spectrum deacylase activity of Sirt4 seems to play a key role to stimulate leucine catabolism *in vivo* and to indirectly inhibit amino acid-stimulated insulin secretion (AASIS) (15).

## Sirt4 Modulation of Lipid Metabolism

Sirt4 plays a pivotal role in the modulation of fatty acid metabolism in both skeletal muscle and white adipose tissue by deacetylating and inhibiting MCD (11) (Figure 1A). In its deacetylated form, MCD no longer catalyzes the conversion of malonyl-CoA into acetyl-CoA, thus causing malonyl-CoA accumulation. Malonyl-CoA serves at the same time as the chain-elongating unit for fatty acid biosynthesis and as an allosteric inhibitor of the fatty acid transporter carnitine palmitoyl-transferase (CPT1), responsible for the transfer of fatty acids from the cytosol to the mitochondrial matrix for β-oxidation. While nutritional rich conditions correlate with accumulated malonyl-CoA and the consequent arrest of the FAO process and increase of fat synthesis, during a fasted state, lower levels of malonyl-CoA have been registered and FAO is increased for energy production (17–19). This regulatory mechanism was confirmed in Sirt4 knock-out (KO) mice that showed raised FAO associated with increased exercise tolerance and resistance to diet-induced obesity (20–22). MTPα is a mitochondrial enzyme that catalyzes two steps of FAO and can be acetylated or ubiquitinated on the same three lysine residues. In hepatocytes, it was demonstrated that the Sirt4-mediated deacetylation of MTPα promotes its ubiquitination and proteasome-dependent degradation, thus contributing to the inhibition of FAO (12). Sirt4 expression is increased in livers of patients with non-alcoholic fatty liver disease (NAFLD) (12, 23). The evidence that decreased Sirt4 expression in mice livers is able to protect against NAFLD by inhibiting MTPα deacetylation and degradation suggested that the upregulated levels of Sirt4 observed in NAFLD patients could contribute to the onset of the disorder by decreasing MTPα-catalyzed FAO and, consequently, by promoting ectopic lipid accumulation (12). Sirt4 can also inhibit FAO in mice liver as well as hepatoma, kidney, and fibroblast cells by repressing the activity of peroxisome proliferator-activated receptor α (PPARα), a transcription factor that stimulates the expression of FAO genes. Indeed hepatocytes from Sirt4 KO mice showed an increased FAO rate, which was dependent on the interaction of Sirt1 with PPARα and on the Sirt1-dependent deacetylation and activation of the transcriptional co-activator peroxisome proliferator-activated receptor gamma co-activator 1-α (PGC-1α). These findings suggest that Sirt4 works in a retrograde signaling pathway from the mitochondria to the nucleus to decrease Sirt1 activity, likely by competing for NAD^+^ (17, 24) (Figure 1A).

AMP-activated protein kinase (AMPK) plays a crucial role in promoting FAO through the phosphorylation and the inhibition of acetyl-CoA carboxylase (ACC), the enzyme that, counteracting MCD, catalyzes the carboxylation of acetyl-CoA to provide malonyl-CoA. In fact, the consequently reduced malonyl-CoA levels correlate with an increased CPT1-mediated mitochondrial fatty acid uptake (17, 25, 26). In addition, AMPK is also known for its capability to act as a transcriptional co-activator of PGC-1α (27). In this context, it is noteworthy that Sirt4 KO mice livers presented elevated levels of activated AMPK, resulting in the inhibition of ACC, the reduction of malonyl-CoA, PGC1-α induction, and the final promotion of FAO (5) (Figure 1A).

## Sirt4 as a Checkpoint Between Glycolysis and TCA Cycle

The mitochondrial multi-protein complex PDH (28) promotes the oxidative decarboxylation of pyruvate to provide acetyl-CoA, thus linking glycolysis and the TCA cycle. Sirt4 can remove lipoyl groups from the PDH subunit dihydrolipoyl lysine-residue acetyltransferase efficiently in a phosphorylation-independent manner, thereby repressing the enzymatic activity of the complex both in cells and *in vivo* (9). Since PDH activity indirectly modulates a large variety of downstream metabolic processes, the PDH regulator Sirt4 can be considered as one of the main regulators of cellular metabolism (Figure 1B).

## Sirt4 Modulation of Insulin Secretion and Sensitivity

Insulin secretion by pancreatic β-cells can be promoted by amino acids or glucose (29). Amino acids are metabolized into TCA cycle intermediates to generate adenosine triphosphate (ATP) that finally stimulates insulin release. Glutamine is initially hydrolyzed by glutaminase (GLS) to glutamate, which is subsequently converted by GDH into the TCA cycle intermediate α-ketoglutarate (30). As mentioned, Sirt4 can catalyze the mono-ADP-ribosylation and inactivation of GDH in pancreatic β-cells (Figure 1C) and, through this mechanism, can contribute to the reduction of ATP levels and the repression of AASIS (8). Indeed pancreatic islets isolated from Sirt4 KO mice showed both increased GDH activity and circulating levels of insulin (8).

Sirt4 can modulate insulin secretion also by other mechanisms (3). Leucine is an allosteric activator of GDH, and Sirt4-promoted leucine catabolism *via* MCCC deacylation and activation will thus prevent GDH activation and result in decreased glutamine-stimulated insulin. Indeed Sirt4 KO mice displayed not only increased glutamine- and glucose-stimulated insulin secretion but also improved leucine-stimulated insulin release. Sirt4 can also interact with the insulin-degrading enzyme (IDE), thus reducing insulin secretion in response to glucose (29) (Figure 1C).

Sirt4 might also promote insulin release by increasing the ATP levels as a result of the deacylation and the inhibition of the uncoupling ADP/ATP carrier protein adenine nucleotide translocator 2 (31) (Figure 1C). Due to its many different activities in inhibiting insulin secretion, Sirt4 has been linked to the emergence of T2D, which was also confirmed by studies in Sirt4 KO mice that quickly develop hyperinsulinemia, insulin resistance, and glucose intolerance (15). Furthermore, Sirt4 protects podocytes against reactive oxygen species (ROS) accumulation and apoptosis under hyperglycemic conditions and thus has a protective role against diabetic nephropathy, one of the worst complications associated with diabetes (32).

## Sirt4 and Mitochondrial Oxidative Stress

ROS are by-products of oxidative metabolism, mainly derived from oxidative phosphorylation and enzymatic reactions (α-glycerophosphate dehydrogenase, NADPH-oxidase, mono-aminoxidase, etc.) in the mitochondria. Since moderate levels of ROS have physiological roles in stress responses and signal transduction, both too high and excessively low ROS levels have significant pathogenic roles (6).

Sirt4 is involved in the regulation of ROS production in the mitochondria, very likely through a scaffolding function (19). Sirt3, another mitochondrial deacetylase, deacetylates, and activates the anti-oxidant enzyme manganese superoxide dismutase (MnSOD) to decrease the mitochondrial ROS levels. Sirt4 can compete with MnSOD for binding to Sirt3, thus sequestering Sirt3, and indirectly inhibiting Sirt3-mediated MnSOD activation (Figure 1D). This action, which seems non-enzymatic because the catalytically inactive mutant of Sirt4 H161Y is also able to block the Sirt3–MnSOD interaction, induces an increase of ROS levels and oxidative stress in the mitochondria of heart muscle cells and promotes cardiac hypertrophy (19). However, as mentioned, the overexpression of Sirt4 can prevent podocyte apoptosis induced by glucose with concomitantly increased mitochondrial membrane potential and reduced ROS production (32). These findings reveal that the effects of Sirt4 on mitochondrial ROS levels are context dependent.

## Sirt4 as a Context-Dependent Tumor Suppressor and Oncoprotein

Most studies on the involvement of Sirt4 in tumor biology highlight this enzyme as a mitochondrion-localized tumor suppressor due to its crucial regulatory role of mitochondrial metabolism during tumorigenesis (5). Sirt4 mRNA levels are reduced in many human cancers, such as lung, pancreatic, ovarian, gastric, colorectal, prostate, renal, liver, and endometrial cancer as well as hematological tumors (33–45) (Table 1). Lower levels of Sirt4 protein expression in tumor tissues are often associated with worse pathological grading and reduced survival in cancer patients, and Sirt4 KO mice display an increased incidence of spontaneous tumors (2, 33, 36, 37, 44, 45, 49).

**Table 1:** Roles of Sirt4 in tumorigenesis.

**Phenotype**	**Cancer types**	**Role of Sirt4**	**References**
Sirt4 downregulation	Acute myeloid leukemia, Burkitt lymphoma, and lung, colorectal, gastric, liver, lung, endometrial, pancreatic, ovarian, prostate, and renal cancers	Cancer suppression: inhibition of tumor proliferation, invasion, and migration	(4, 33–35, 37–46)
Sirt4 over-expression	Esophageal cancer, hepatocarcinoma (HepG2 cells)	Cancer promotion: enhanced cancer cell survival rate in “extreme” DNA-damaging conditios	(4, 47, 48)

While quiescent cells exploit the TCA cycle to obtain energy from glucose, proliferating cells mainly use it as a carbon source for lipogenesis through the mitochondrial efflux of citric acid. This efflux needs to be replaced by an influx of TCA cycle intermediates, known as anaplerosis, and glutamine is the main source for TCA anaplerosis in proliferating cells (50) (Figure 1E). Therefore, glutamine catabolism plays a crucial role for the proliferation of tumor cells by replenishing TCA cycle intermediates to support increased growth and by generating ammonia, which neutralizes the acidic metabolites usually produced because of the increased glycolysis in tumor cells (Figure 1E). In addition, glutamine by itself promotes the activation of the tumor suppressor p53, which is responsible for apoptosis and tumor regression induction (51) (Figure 1E). For these reasons, Sirt4 repression of mitochondrial glutamine catabolism by the inhibition of GDH activity seems to contribute significantly to its function as a tumor suppressor.

Many studies have supported this notion, providing mechanistic insights into the relation between glutamine metabolism and Sirt4 dysregulation during tumorigenesis (3). *c*-Myc is an oncogenic transcription factor that can increase the expression of GLS *via* the repression of specific microRNAs (miR-23a and miR-23b) and consequently stimulates glutamine metabolism in many *c*-Myc-driven cancers (38), which typically show marked glutamine dependence (52, 53). Sirt4 can inhibit the proliferation of *c*-Myc-induced Burkitt lymphoma by inhibiting GDH activity, thereby preventing increased glutamine metabolism and sensitizing it to glucose depletion (38) (Figure 1E). Both the transcriptional regulator *C*-terminal binding protein (CtBP) and the mammalian target of rapamycin complex 1 (mTORC1) (54) were discovered to promote glutamine metabolism and tumor proliferation in various cancer contexts by repressing the expression of Sirt4 directly (55) and indirectly (54), respectively (Figure 1E).

In addition to the regulation of mitochondrial glutamine catabolism, Sirt4 also has the capability to modulate other cancer-related cellular features such as cell cycle progression, apoptosis, invasion, and metastatic potential. It is noteworthy that these processes, in many cases, are somehow linked to the effects on glutamine metabolism (56). Sirt4 expression is upregulated by different types of DNA damage. It can prevent cell cycle progression following DNA damage (37), and Sirt4-mediated inhibition of glutamine anaplerosis can be crucial for a productive cell cycle arrest upon DNA damage (56) and for the proper implementation of the cellular DNA damage repair program (6, 37). Moreover, in colorectal cancer (CRC) cells, Sirt4 overexpression induces anti-proliferative effects and increases the sensitivity to the drug 5-fluorouracil (46). Sirt4 can also display pro-apoptotic effects in lung cancer cells by inhibiting mitochondrial fission, which is known to prevent apoptosis and enhance cancer cell growth (44). Sirt4 is also able to prevent the epithelial–mesenchymal transition (EMT) in CRC cells by upregulating the expression of E-cadherin, which promotes cell–cell adhesion and consequently inhibits tumor invasion and metastasis (35) (Table 1). It is noteworthy that this upregulation seems to correlate with the Sirt4-mediated inhibition of glutamine metabolism since α-ketoglutarate, the product of the GDH-catalyzed reaction inhibited by Sirt4, decreases E-cadherin expression (35).

However, a few recent studies have demonstrated that Sirt4 can also be upregulated in some tumors and can exert oncogenic properties by regulating the stress responses of cancer cells and preparing them to gain selective advantages, including resistance to anti-tumor treatments (2, 4). Indeed Lai and coworkers showed that the Sirt4 levels in esophageal squamous cell carcinoma (ESCC) tissues from Chinese patients were higher than those in adjacent esophageal normal tissues and that the levels of the protein correlate inversely with the mean survival time of ESCC patients (48) (Table 1). In another report, Sirt4 overexpression in HepG2 cells correlated with reduced levels of cleaved caspase-3, thus reducing apoptotic cell death (anti-apoptotic effect) and increasing the survival and the cellular clone formation rate of tumor cells in response to different DNA-damaging conditions such as cisplatin, radiation, and UV irradiation, and Sirt4 loss sensitized cells to drug treatment (47) (Table 1). Therefore, as far as it concerns the modulation of genome stability, Sirt4 plays a dual role in inhibiting and promoting tumor formation. In fact, while in non-cancer cells Sirt4 protects against the accumulation of DNA damage and promotes DNA repair by acting as a mitochondrial tumor suppressor, it seems to play a different role in cancer cells depending on the external conditions. In “normal” conditions, Sirt4 can inhibit tumor proliferation *via* the repression of the mitochondrial glutamine catabolism and the subsequent reduction of tumor metabolism. In contrast, in “extreme” DNA-damaging conditions, including chemotherapy, Sirt4 can protect tumor cells, allowing them to escape apoptotic death and, potentially, to acquire more mutations and become more aggressive (4) (Table 1).

It is well-known that gene products known for their tumor-suppressive role can act as oncoproteins in relation to the specific (epi)genetic context, tumor type, tissue or organ microenvironment, stage of tumor development, and external stimuli. Further studies thus are necessary to define the role of Sirt4 and the exact mechanisms involved in the balance between anti-stress (anti-apoptosis) and tumor inhibition (pro-apoptosis) under different pathophysiological and pharmacological conditions.

## Conclusion

In the last few years, many studies about Sirt4 have only started to shed light on the multiple enzymatic activities and the biological functions of this, for a long time, enigmatic sirtuin. It is now evident that several Sirt4 actions and substrates are tissue specific, and it is likely that additional enzymatic and non-enzymatic activities and substrates still have to be identified. For example, while Sirt4 associates with different biotin-dependent carboxylases, including MCCC, and is able to hydrolyze various lysine-biotinylated peptides *in vitro*, the capability to remove biotinyl groups from substrate proteins *in vivo* has not been investigated yet (3). In humans, Sirt4 is a key regulator of several mitochondrial metabolic processes (Figure 1) including fatty acid and branched-chain amino acid metabolism, carbon entry into the TCA cycle, electron transport and ROS production, insulin secretion and sensitivity, apoptosis, ATP homeostasis, and redox state responding to nutrient condition and exercise. Moreover, Sirt4 has been indicated as a mitochondrion-localized tumor-suppressor protein that, like other sirtuins, may also act as an oncoprotein depending on the specific tumor type, stage, and overall biological context (Table 1). A better mechanistic understanding of how Sirt4 contributes to tumorigenesis, and more generally, to age-related diseases is certainly necessary to provide new insights for the potential targeting of this enzyme for therapeutic scopes.

## Author Contributions

DT, CS, AM, and DR contributed to conception, manuscript writing, and proofreading. DT and DR searched the literature and prepared the figure about the biological roles of Sirt4. DR supervised and coordinated the whole writing work. All the authors have read and approved the final manuscript.

### Conflict of Interest

The authors declare that the research was conducted in the absence of any commercial or financial relationships that could be construed as a potential conflict of interest.
